# Notes and Notices

**Published:** 1897-09

**Authors:** 


### NOTES AND NOTICES.

Dr. Jean Ida MacKay Gliddon has just returned to the United States after a prolonged sojourn in Europe, particularly London, and will resume the practice of medicine at her former residence, Butte, Montana.

Dr. M. R. Faulkner has just received his appointment from Washington as Pension Examiner to fill the position on the Cumberland County Board made vacant by the death of Dr. Wiley. This is an important appointment, and Dr. Faulkner is receiving the congratulations of his many friends.—*The Evening Journal, of Vineland, N. J.*

Eye-sight Testing.—It gives us great pleasure to call the attention of the profession to the picture and advertisement in the present issue. We cordially recommend Mr. Martin, the optician, to the profession, as he makes examination and measurement of the eyes for glasses without the use of Atropine. We have had occasion to send him our own patients for several years past with much satisfaction.

Dr. Joseph Hasbrouck, of Dobbs Ferry, on the Hudson, was badly injured recently by his team running away while he was out on his round of professional visits. The coachman was rendered insensible by his fall from the vehicle, and one horse was killed.

### FUN FOR DOCTORS.

### FOOD AND MEDICINE.

There was a Law-Doctor called Leverson;
A Gentleman sure, or there never's none;
A strong single-tax
And number one Anti Vax,
But he mingled his food with his medicine.

F. P.

(See Dr. Leverson's article "Calcarea and the Proteins," in The Homœopathic Physician for July, 1897, page 283.)

Quack, Quack.—Mamma: Look, daughter, dear, here is the doctor coming! What a favorite he is. See, even the little chickens run to meet him!

Daughter: Yes, Mamma, and the little ducks cry, "quack, quack!"

When All Men Are Doctors.—"Brooks," said Rivers, "you ought to do something for that cold of yours. A neglected cold sometimes leads to serious consequences.

"This cold of mine isn't neglected," crossly answered Brooks. "Five or six hundred of my friends are looking after it."—*Chicago Tribune.*

